# Intensified secondary prevention intending a reduction of recurrent events in TIA and minor stroke patients (INSPiRE-TMS): a protocol for a randomised controlled trial

**DOI:** 10.1186/1471-2377-13-11

**Published:** 2013-01-24

**Authors:** Stefanie Leistner, Georg Michelson, Inga Laumeier, Michael Ahmadi, Maureen Smyth, Gabriele Nieweler, Wolfram Doehner, Jan Sobesky, Jochen B Fiebach, Peter Marx, Otto Busse, Friedrich Köhler, Holger Poppert, Martin LJ Wimmer, Thomas Knoll, Paul Von Weitzel-Mudersbach, Heinrich J Audebert

**Affiliations:** 1 Department of Neurology, Charité – Universitätsmedizin Berlin, Campus Benjamin Franklin, Hindenburgdamm 30, 12203, Berlin, Germany; 2 Department of Ophthalmology, Universitätsklinikum Erlangen, Maximiliansplatz 2, 91054, Erlangen, Germany; 3 Center for Stroke Research Berlin, Department of Neurology, Charité – Universitätsmedizin Berlin, Campus Virchow-Klinikum, Augustenburger Platz 1, 13353, Berlin, Germany; 4 Center for Stroke Research Berlin, Department of Neurology, Charité – Universitätsmedizin Berlin, Campus Berlin Mitte, Charitéplatz 1, 10117, Berlin, Germany; 5 Center for Stroke Research Berlin, Department of Neurology, Charité – Universitätsmedizin Berlin, Campus Benjamin Franklin, Hindenburgdamm 30, 12203, Berlin, Germany; 6 Generalsekretaer Deutsche Schlaganfall-Gesellschaft, Reinhardtstrasse 27C, 10117, Berlin, Germany; 7 Center for Cardiovascular Telemedicine, Charité – Universitätsmedizin Berlin, Campus Berlin Mitte, Charitéplatz 1, 10117, Berlin, Germany; 8 Department of Neurology, Technische Universität München, Klinikum rechts der Isar, Ismaninger Str. 22, 81675, München, Germany; 9 Gemeinschaftspraxis Dr.med.Thomas Knoll, Martin L.J.Wimmer und Dr. med. Michael Oppenheimer, Prinzregentenplatz 13, 81675, München, Germany; 10 Department of Clinical Medicine - The Department of Neurology, Nørrebrogade 44, 8000, Aarhus C, Denmark

**Keywords:** TIA, Stroke, Secondary prevention, Intervention trial

## Abstract

**Background:**

Patients with recent stroke or TIA are at high risk for new vascular events. Several evidence based strategies in secondary prevention of stroke are available but frequently underused. Support programs with multifactorial risk factor modifications after stroke or TIA have not been investigated in large-scale prospective controlled trials so far. INSPiRE-TMS is a prospective, multi-center, randomized open intervention trial for intensified secondary prevention after minor stroke and TIA.

**Methods/design:**

Patients with acute TIA or minor stroke admitted to the participating stroke centers are screened and recruited during in-hospital stay. Patients are randomised in a 1:1 ratio to intervention (support program) and control (usual care) arms. Inclusion of 2.082 patients is planned. The support program includes cardiovascular risk factor measurement and feedback, monitoring of medication adherence, coaching in lifestyle modifications, and active involvement of relatives. Standardized motivational interviewing is used to assess and enhance patients’ motivation. Primary objective is a reduction of new major vascular events defined as nonfatal stroke and myocardial infarction or vascular death. Recruitment time is planned for 3.5 years, follow up time is at least 2 years for every patient resulting in a total study time of 5 years (first patient in to last patient out).

**Discussion:**

Given the high risk for vascular re-events in acute stroke and the available effective strategies in secondary prevention, the INSPIRE-TMS support program has the potential to lead to a relevant reduction of recurrent events and a prolongation of the event-free survival time. The trial will provide the basis for the decision whether an intensified secondary prevention program after stroke should be implemented into regular care. A cost-effectiveness evaluation will be performed.

**Trial registration:**

clinicaltrials.gov: 01586702

## Background

Patients with recent stroke or TIA are at high risk for a recurrent stroke or other major vascular events [1-4]. Despite this high risk and the availability of evidence based secondary prevention strategies, risk factor control and medication adherence frequently remain under recommended targets [5].

This observation has a variety of reasons: Although at much higher risk, post-stroke patients do often not receive more intensive medical care than patients in primary prevention [4,6]. The separation of health systems in hospital-based and outpatient care as in Germany is a major barrier for optimal secondary prevention as recommendations given in the acute stroke facility are frequently not followed by primary care physicians (in part depending on their specialty) [4]. Another problem is that despite a high motivation for optimal secondary prevention shortly after experiencing the initial cerebrovascular event, many patients don’t stick with the recommended measures over a longer time period. One-third of stroke patients discontinue secondary prevention medication [7] and control of arterial hypertension as the most important risk factor for stroke remains unsatisfactory across countries [4,8].

Positive experiences with structured support programs have been gained in other diseases such as diabetes mellitus [9], coronary heart syndrome [10,11] and arterial hypertension [12] yielding better quality of risk factor control and remarkable reductions in clinical event rates [10,13].

Considering the typical stroke and TIA population which consists mainly of elderly patients frequently having multiple risk factors and a high risk for dependency after a recurrent event, a structured supported secondary prevention program after stroke appears promising. However, it has never been tested in an adequately powered trial. As a consequence, stroke has not been selected for chronic disease management programs.

A recent observation strengthens the hypothesis that patients with cerebrovascular disease are likely to benefit from a multimodal secondary prevention scheme. Intensified medical treatment in patients with symptomatic arterial stenosis (forming the control arm and compared against stenting) was associated with a more than 50% lower recurrence rate in the SAMMPRIS trial [14] compared to patients with the same inclusion criteria but non supported secondary prevention in the WASID trial [15]. However this effect was not observed in a face-to-face trial but in a comparison with a historical cohort.

The INSPiRE-TMS trial is therefore designed to evaluate the effects of a structured support program focussing on cardiovascular risk factor control, monitoring of medication adherence, coaching in lifestyle modifications, and active involvement of relatives over a period of at least 2 years.

## Methods/design

### Study design

INSPIRE is a prospective, multi-center, randomized open intervention trial for intensified secondary prevention initiated and coordinated by the Center for Stroke Research Berlin. Approval was obtained by the local ethics committee (EA2/084/11). The study has been registered at the trial registration (clinicaltrials.gov: 01586702).

### Objectives

*Primary objective:* With regard to new vascular events the study shall prove that the participation in a patient-centered intensified secondary prevention program increases the event-free survival time during follow up compared to the participation in usual care.

Secondary objectives:

To improve risk factor control and adherence to medical recommendations

To evaluate the effect of optimized secondary prevention on surrogate parameters such as physical fitness, vascular changes on retinal fundus and silent vascular lesions in MRI

To evaluate the influence of body weight and other metabolic parameters on vascular event rates in secondary prevention

To calculate the cost-effectiveness of the support program.

### Participants

Acute patients with TIA (clinical restitution within 24 hours and ABCD2-Score ≥3 or visible DWI-lesion in MRI) or minor stroke (mRS ≤ 2 at time of screening and visible DWI-lesion in MRI) evaluated in a dedicated and organized setting of care (Stroke Unit, out-patient clinic) in Germany and Denmark will be included in the study. Participating study centres are the Departments of Neurology at the Charité-University Medicine Berlin, Campus Benjamin Franklin, Campus Virchow-Klinikum and Campus Mitte, the Department of Neurology, Klinikum rechts der Isar at the Technische Universität of Munich, the Department of Neurology at the Klinikum Ludwigshafen (all in Germany), and the Department of Neurology at the University of Aarhus, Denmark.

*Inclusion criteria:* Age over 18 years; acute stroke or TIA within 14 days prior to inclusion into the study; at least one of the following risk factors: arterial hypertension, diabetes mellitus, atrial fibrillation and/or smoking; written informed consent, and realistic perspective in keeping the outpatient appointments.

*Exclusion criteria*: Distance from home to study center not in suitable range for keeping the outpatient appointments, cognitive impairment jeopardizing adherence to the support program, modified Rankin Score >2 at time of study inclusion, malignant disease with life expectancy of less than 3 years, relevant alcohol or other substance abuse (except for nicotine), stroke or TIA aetiology without options for evidence based secondary prevention (e.g. dissection or vasculitis).

### Recruitment and randomization

Consecutive acute patients with TIA or minor stroke admitted to the Stroke Units or TIA clinics of the participating centers will be included during in-hospital or in-clinic stay. Patient recruitment is planned over a 3.5-years period. Follow-up is planned for a minimum of 2 years for every patient after study inclusion in order to complete the 2 years intervention program in all patients. Therefore the study time from first patient in to last patient out is 5 years in total.

After approval from the participants and completion of the initial assessment, patients will be formally entered into the study and randomized with a 1:1 randomization to intervention and control arm by a web-based online randomization procedure provided by the Coordination Center for Clinical Studies (KKS) at the Charité.

### Procedures

**Figure 1:**
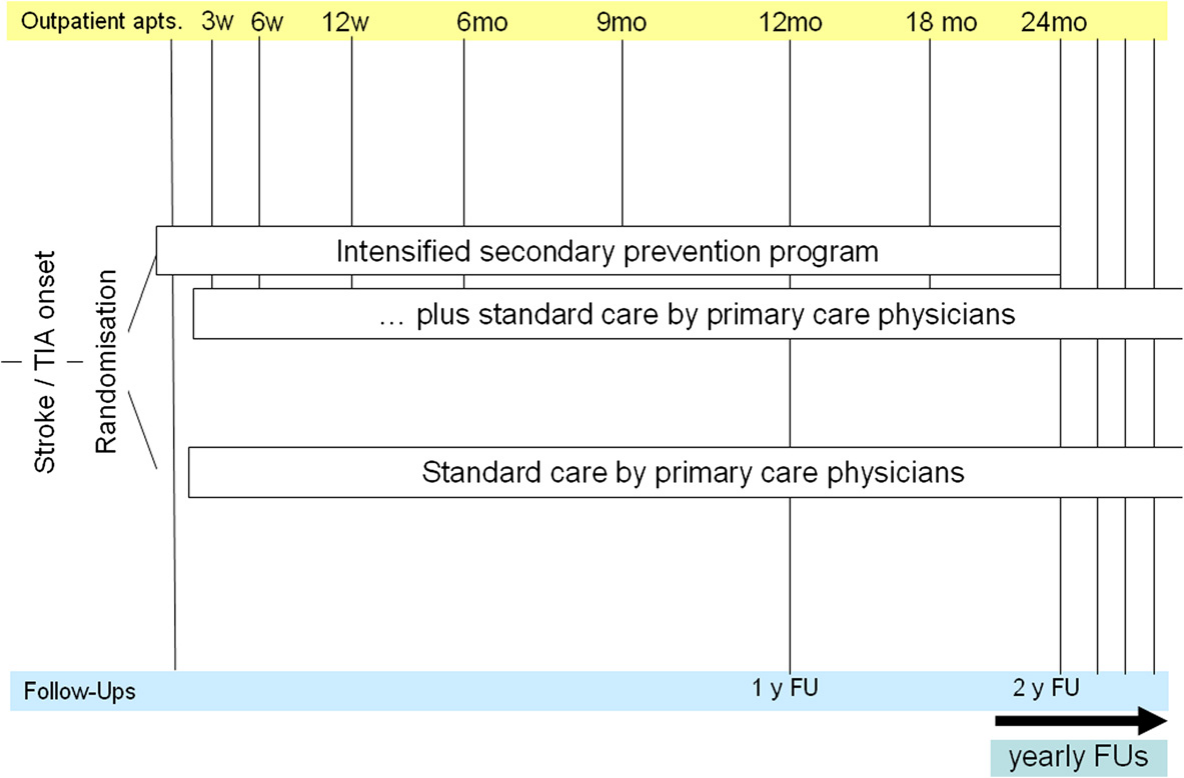
Flow chart of study procedures.

Study procedures are depicted in the flow chart (Figure 1).

*Standardized baseline assessment* for intervention and control groups will include a questionnaire regarding demographic information, risk factors and co-morbidities, clinical symptoms of the acute cerebrovascular event, and duration of symptoms.

*Standard clinical evaluation* will consist of a neurological examination according to the National Institutes of Health Stroke Scale (NIHSS) at admission and modified Rankin Scale (mRS) as well as Barthel Index (BI) at time of study inclusion. Stroke aetiology will be classified according to TOAST criteria [16]. Laboratory measures will include LDL cholesterol, CRP, HbA1c, and INR. Body mass index (BMI, kg/m2) will be calculated from height and weight measurements. Blood pressure will be measured manually in sitting patients (resting at least 5 min) on both arms. Both blood pressure values will be recorded and the higher value will be used for statistical comparisons. Arterial hypertension is defined by either repeated elevated systolic blood pressure >140 and/or diastolic blood pressure >90 mm Hg or the previous use of antihypertensive drugs. Diabetes is defined by either HbA1c ≥6.5% or use of antidiabetics. Hyperlipidemia is defined by either LDL cholesterol > 100 mg/dl or use of lipid-lowering drugs. Smoking will be evaluated in number of cigarettes per day and in pack years. Patients who currently don’t smoke will be divided in non-smokers and past smokers (within the last 5 years). The time since smoking cessation will be documented. Physical activity is measured as frequency of “physical activity with an intensity leading to transpiration and/or elevated breathing frequency over more than 30 min” per week. Detailed and structured information concerning vascular risk factor targets will be given to the patients before discharge and these recommendations will be included in the discharge letter to primary care physicians (PCP) irrespective of the trial allocation.

*Standard care* will consist of outpatient care usually guided by a primary care physician. The treating primary care physician will be informed about the results of risk factor measurements at the yearly follow-up appointments but no further recommendations will be given to the patient or PCP by the study team.

#### Intervention program

The target values for secondary prevention are orientated at the recommendations of the German Society of Neurology respective the European Stroke Organisation. For patients with high recurrence risk in large vessel disease (see above), targets are adapted on the basis of “aggressive medical treatment” of the SAMMPRIS trial [14].

The intensified support program is planned with a total of eight outpatient appointments. The structured assessment will include risk factor control, medication intake, compliance with oral anticoagulation therapy (including monitoring of INR in Vitamin K antagonist treatment) and joint agreement on an individual target plan.

Target values for risk factors are:

Blood pressure <140/85 mmHg (<130/80 in diabetic patients) [16]

Physiological circadian blood pressure with >10% lower BP values during nighttime

HbA1c <7.5%

Nicotine abstinence

LDL <100 mg/dl in patients with arterial or cardioembolic stroke without specifically elevated risk, LDL ≤70 mg/dl with CHD, LDL ≤70 mg/dl in patients with symptomatic intracranial stenosis judged as hemodynamically relevant (independent from examination technique) or extracranial stenosis >50% according NASCET criteria without surgical intervention, LDL ≤70 mg/dl in patients with evidence of instable plaque (in plaque imaging) in the corresponding extracranial artery

Physical activity ≥30 min per day at least three times per week (oriented at the recommendations of the German Society of Neurology

Healthy nutrition (intake of 4–5 portions of fruit and/or vegetables per day)

The targets for pharmaceutical treatment are:

Platelet inhibitors for all patients with Stroke/TIA caused by small or large vessel disease

Dual antiplatelet therapy over three months in patients with symptomatic intracranial stenosis judged as hemodynamically relevant (independent from examination technique) or extracranial stenosis >50% (according NASCET) without surgical intervention or in patients with evidence of instable plaque (in plaque imaging) in corresponding extracranial artery

Oral anticoagulation (INR 2–3) or regular intake of new oral anticoagulants in patients with atrial fibrillation

Statin treatment (Simvastatin 40 mg or Atorvastatin 80 mg) in patients with LDL >100 mg/dl or LDL ≤70 mg/dl in patients with high recurrence risk.

Intervention strategies:

Comprehensive and repeated information about pathophysiology of the individual risk for recurrent event of stroke or TIA and potentials of vascular risk reduction. This information will be offered to the patients during in-hospital stay but also as part of the outpatient appointments. Next of kin will be involved as much as possible.

An individual plan regarding target values and medication will be agreed upon with the patient using methods of motivational interviewing

The patient’s motivation will be enhanced using feed-back-strategies regarding measured risk factors, target measurements (e.g. INR) and “memory parameters” such as HbA1c or physical fitness

Complementary offers: assistance in finding peer groups and group therapies (e.g. nordic walking, INR self measurement and smoking cessation programs).

Required certificates and training for involved professionals and treatment algorithms:

Patients will receive counseling by board certified physicians with special training in neurovascular medicine at all intervention appointments. Preparation for medical counseling by assessment of risk factor control can be performed by trained nurses or healthcare assistants.

A standardized motivational interviewing training over 2 days and reinforcement training over one day after approximately 6 months is compulsory for all physicians involved in counseling during appointments in the support program.

Standard operating procedures are used for concrete action in case of critical deviations from target values regarding blood pressure and anticoagulation.

Information about risk factor control is communicated to family doctors via standardized reports including telephone conversation in case of urgently needed action or aimed interdisciplinary consensus.

### Study endpoints

#### Primary endpoint

Composite of non fatal stroke (including strokes with new tissue-based definition), non fatal major coronary event and cardiovascular death.

Secondary endpoints:

Rate of patients who meet the recommended guideline targets regarding blood pressure, smoking cessation, serum-LDL, physical activity, HbA1c (in patients with diabetes), therapeutic range of INR or regular intake of new oral anticoagulants in patients with atrial fibrillation,

Effects on vascular surrogate parameters (microalbuminuria, physical fitness,)

Total mortality

Frequency of hospital admissions for vascular diseases (including TIA, angina pectoris with vascular interventions, peripheral arterial occlusive disease with vascular intervention)

Number of days “alive and at home”.

#### *Substudy endpoints*

Effects on vascular surrogate parameters (intima-media-thickness status of retinal fundus “cerebrovascular lesion load” in MRI), effects on quality of life and mood, cost effectiveness, correlation between risk factor control, surrogate parameters and biomarkers (exploratory).

### Power and sample size calculation

The expected event rate in the control arm was estimated on the basis of the results of more recently published studies [2,3,6,17]. The expected relative risk reduction was calculated in a conservative way only on the basis of blood pressure reductions and irrespective of additional effects of other risk factor modifications.

The effect size (relative risk reduction) was estimated on the basis of the observed effects of blood pressure reduction in recent secondary prevention trials [1,18] and the mean blood pressure differences between two consecutive cohorts evaluated in the Charité hospitals in Berlin [19]. The first cohort was observational and represented standard care in Berlin. The second cohort underwent a stepwise modeled support program that included up to four outpatient appointments over a period of 6 months, using the same strategies as described above. The basis of the sample size estimation is an expected event rate of 16.94% after three years in the control group and a relative risk reduction of 28% in the intervention group resulting from relative risk reductions per mmHg in other secondary prevention studies. The sample size was calculated assuming an aimed power of 80%. After consideration of a non-adherence rate of 10% in the intervention group and a drop-out rate of 5% in both arms, the case number results in 2.082 patients (1.041 patients per group).

After analysis of mean blood pressure differences in the 1-year follow-up of the first 500 patients, the expected effect size and the power calculation will be re-adjusted.

### Statistical analysis

The primary analysis is a time to event analysis using Cox-regression-models. An interim analysis after 160 observed events will be done by a statistician blinded to the study arm. The interim analysis will indicate whether the sample size and study duration has to be modified according to the overall observed event rate. The sample size needs not to be adjusted for this blinded interim analysis as only the overall event rate will be considered. The assessment of the true drop out rate will be part of the (blinded) interim analysis. The dichotomized secondary endpoints will be evaluated according to the primary outcome measure. Continuous variables will be analyzed with the appropriate tests. The clustering of the data by the different centres will be accounted for by using frailty models [20].

### Methods against BIAS

Follow-up assessment are planned to be done by study nurses not involved in providing the individual support program for the patient aiming to keep them blinded to the study allocation. Endpoints will be assessed in a written questionnaire that will be handed out to the patients prior to further follow-up assessments.

The primary endpoints and serious adverse events will be verified and adjudicated by the Endpoint and SAE Adjudication Committee that is blinded to the trial allocation of the individual patients.

### Ethical considerations

The trial does not include any experimental treatments but compares usual care with more intensive care based on national and international guidelines aiming at an improved adherence to evidence based recommendations. The study has been approved by the Ethics Committee of the Charité (EA2/084/11). Patients are insured against travel accidents.

## Discussion

Depending on aetiology, the natural risk for stroke recurrence during the first year after event has been described between 5% [2] (stroke patients without high risk factors) and 20% [15,21-24]. A secondary ischemic event has considerably more serious consequences leading to physical disability in more than 60% and death in more than 20% [25]. In addition to the medical and personal impact of the recurrent stroke, the costs of recurrent events and rehospitalisation are enormous. Lifetime costs per first ever stroke case are more than 40.000 Euro in Germany [26].

Growing evidence suggests that early diagnostic work-up with consequent initiation of multimodal preventive measures results in a major reduction of re-events. In patients with TIA, the “effect of urgent treatment of transient ischemic attack and minor stroke on early recurrent stroke” (EXPRESS) study was conducted as a population based sequential comparison [27]. It demonstrated that urgent assessment of stroke aetiology and early start of secondary prevention was associated with reduced risk for recurrent stroke. The interventions comprised immediate start of poly-pharmaceutical risk factor control, antithrombotic therapy and interventional treatment for symptomatic carotid stenosis. However, patients were followed-up for only three months. Very similar effects were found in the SOS-TIA study with much lower recurrence rates as expected according to the ABCD [2] score [17].

Hence, it seems of crucial importance to start a secondary prevention program at an early stage after the cerebrovascular event. This is particularly important because shortly after the index event, patients are generally very motivated for participation in a structured support program.

In contrast to other diseases such as diabetes mellitus, coronary heart disease and COPD, stroke has not been selected for chronic disease management programs in Germany. Therefore, neither hospitals nor primary care physicians are able to offer intensive monitoring of cardiovascular risk factors to patients after stroke or TIA. Recommendations given in discharge letters frequently focus on medical treatments but not on recommendations regarding non-pharmacological treatments such as life style modifications including physical activities and dietary aspects. Family members are usually not involved in the treatment concepts. Since lifestyle is strongly influenced by the social network, patients frequently continue the unhealthy lifestyle. Smoking, poor diet coupled with physical inactivity and alcohol consumption are still leading causes of mortality [28].

While the primary objective of INSPiRE-TMS is to investigate the effects of a structured multifactorial support program on the vascular re-event-free survival time, a number of completed or ongoing randomized studies examine effects of supported secondary prevention on differences in risk factor control, medication adherence or surrogate parameters. To our knowledge, none of them has been adequately powered to detect a difference in vascular recurrence free survival time [29-38].

Some limitations of the study will need to be considered: First, there is a potential contamination effect in patients who are randomized to the control group. With the information given before randomization and during follow-up visits, these patients may be better informed about effective vascular prevention compared to the normal post-stroke population. This effect is frequently seen in prospective controlled trials. However, it seems unlikely that this contamination will lead to the same effect as the structured intervention program. Second, the information about life style parameters, adverse events and endpoint events will be self-reported. Thus, self-reported errors are to be expected in both groups and expectation bias by study personnel may occur in follow-up documentation. In order to control for these biases, follow-up assessment is planned to be done by study nurses blinded to the trial arm and not involved in the individual support program. The primary endpoints and serious adverse events will be documented in a written form by the patient prior to the follow-up-interview and will be adjudicated by a blinded Endpoint Committee. Third, the study is conducted in two European health systems and generalizability of the study results may therefore be questioned. However, the methods of intervention are standardized as far as possible in individual counseling and both the quality of risk factor control and rate of recurrent events will be described in detail in the control and intervention arms. This should allow a projection of the study results into other health systems.

In summary, the INSPiRE-TMS trial is designed to answer the question whether a structured support program for secondary prevention after a cerebrovascular event is effective in reducing new major vascular events. The results of the trial are expected for the year 2016.

## Abbreviations

BI: Barthel Index; BP: Blood pressure; BMI: Body Mass Index; CHD: Coronary Heart Disease; COPD: Chronic Obstructive Pulmonary Disease; CRP: (C Reactive protein); DWI: Diffusion-Weighted Imaging; HbA1c: Hemoglobin A 1c; INR: International Normalized Ratio; INSPiRE-TMS: Intensified Secondary Prevention Intending a Reduction of Recurrent Events in TIA and Minor Stroke Patients; KKS: Center for Clinical studies (Zentrum fuer Klinische Studien); LDL: Low density lipoprotein; MRI: Magnetic Resonance Imaging; MOCA: Montreal cognitive assessment; mRS: Modified Rankin scale; NASCET criteria: North American Symptomatic Carotid Endarterectomy Trial criteria; NIHSS: National Institutes of Health Stroke Scale; PCP: Primary care Physician; SAE: Serious adverse event; TIA: Transient ischemic attack; TOAST criteria: Trial of ORG 10172 in Acute Stroke Treatment criteria.

## Competing interests

The authors declare that they have no competing interests.

## Authors’ contributions

HJA conducted the design of the trial. SL, WD, JS, OB, FK were involved in further planning. HJA, SL drafted the manuscript. IL, MS, MA, GN, HP, MW, TK will recruit patients. HJA will participate in the supervision of the trial. GM investigates status of retinal fundus, JF is responsible for MRI lesion load measurements. PM, OB, FK: endpoint committee. All authors approved the final manuscript.

## Funding

The project has received funding from the Center for Stroke Research Berlin (CSB) (01 EO 0801), German Federal Ministry of Education and Research (BMBF) and Berlin Technology Foundation. Prof. Dr. H.J. Audebert received honoraria payment from Lundbeck Pharma, Boehringer Ingelheim Pharma, Takeda Pharma, Sanofi Synthelabo, Bayer Vital, UCB Pharma, Pfizer and BMS. JBF reports receiving consulting, lecture and advisory board fees by BMS, Siemens, Philips, Perceptive, Bio-Imaging Technologies, Boehringer Ingelheim, Lundbeck and Sygnis.

## Pre-publication history

The pre-publication history for this paper can be accessed here:

http://www.biomedcentral.com/1471-2377/13/11/prepub
